# Maternal Immunization Against Respiratory Syncytial Virus: An Updated WAidid Consensus Document on Evidence, Implementation, and Public Health Priorities

**DOI:** 10.3390/vaccines14090768

**Published:** 2026-09-02

**Authors:** Susanna Esposito, Matteo Riccò, Bahaa Abu-Raya, Giancarlo Icardi, Vana Spoulou, David Greenberg, Oana Falup Pecurariu, Ivan Fan-Ngai Hung, Albert Osterhaus, Vittorio Sambri, Nicola Principi

**Affiliations:** 1 Pediatric Clinic, Department of Medicine and Surgery, University of Parma, 43126 Parma, Italy; 2 Servizio di Prevenzione e Sicurezza Negli Ambienti di Lavoro (SPSAL), AUSL-IRCCS di Reggio Emilia, Via Amendola 2, 42122 Reggio Emilia, Italy; mricco2000@gmail.com; 3 Canadian Center for Vaccinology, Dalhousie University, IWK Health Centre and the Nova Scotia Health Authority, Halifax, NS B3K 6R8, Canada; bh723616@dal.ca; 4 Departments of Pediatrics, Dalhousie University, Halifax, NS B3K 6R8, Canada; 5 Departments of Microbiology and Immunology, Dalhousie University, Halifax, NS B3H 4R2, Canada; 6 Department of Health Sciences (DISSAL), University of Genoa, 16132 Genoa, Italy; giancarlo.icardi@unige.it; 7 IRCCS Ospedale Policlinico San Martino, 16132 Genoa, Italy; 8 Immunobiology and Vaccinology Research Laboratory and Infectious Diseases Department “MAKKA”, First Department of Paediatrics, “Aghia Sophia” Children’s Hospital, Athens Medical School, 11527 Athens, Greece; vspoulou@med.uoa.gr; 9 Pediatric Infectious Diseases Unit, Soroka University Medical Center, Faculty of Health Sciences, Ben Gurion University, Beer Sheva 8410501, Israel; dudi1@bgu.ac.il; 10 Children’s Clinical Hospital Brasov, 500063 Brasov, Romania; oanafp@yahoo.co.uk; 11 Faculty of Medicine Brasov, Transilvania University, 500019 Brasov, Romania; 12 Division of Infectious Diseases, Department of Medicine, Queen Mary Hospital, The University of Hong Kong, Hong Kong SAR 999077, China; ivanhung@hku.hk; 13 Research Center for Emerging Infections and Zoonoses, University of Veterinary Medicine Hannover, 30559 Hannover, Germany; albert.osterhaus@tiho-hannover.de; 14 Unit of Microbiology, The Greater Romagna Area Hub Laboratory, 47522 Cesena, Italy; vittorio.sambri@unibo.it; 15 Department Medical and Surgical Sciences (DIMEC), Alma Mater Studiorum University of Bologna, 40126 Bologna, Italy; 16 Department of Pathophysiology and Transplantation, Università degli Studi di Milano, 20122 Milan, Italy; nicola.principi@unimi.it

**Keywords:** respiratory syncytial virus, RSV, maternal immunization, maternal vaccination, infant protection, lower respiratory tract infection

## Abstract

Background: Respiratory syncytial virus (RSV) is a major cause of lower respiratory tract disease and hospitalization in early infancy. The availability of maternal RSVpreF vaccination and long-acting infant monoclonal antibodies has created two effective but operationally distinct pathways for passive protection. Methods: This WAidid consensus document focuses on maternal RSV immunization within an integrated early infancy prevention strategy. A multidisciplinary Consensus Development Group reviewed evidence on infant RSV burden and seasonality, maternal vaccine efficacy and effectiveness, transplacental antibody transfer, long-acting monoclonal antibodies, implementation, equity, and economic considerations. Recommendations were developed using a modified Delphi process with a prespecified consensus threshold of at least 75% agreement. Results: Maternal vaccination can provide protection from birth when administered sufficiently before delivery, whereas direct infant monoclonal antibody prophylaxis provides rapid protection independent of maternal immune response and placental transfer. The relative value of the two approaches depends on gestational age, vaccination-to-delivery interval, infant risk, birth timing, local RSV circulation, antenatal care access, product availability, and cost. Post-pandemic disruption of RSV seasonality increases the importance of flexible strategies that protect infants born outside historically defined seasonal windows. Direct head-to-head evidence remains limited; therefore, policy decisions should integrate trial efficacy, emerging real-world effectiveness, implementation feasibility, and local epidemiology rather than assume universal superiority of one strategy. Conclusions: Maternal vaccination and infant monoclonal antibodies should be positioned within a coordinated prevention pathway. Maternal vaccination may serve as the principal strategy for appropriately timed pregnancies with reliable antenatal access, while infant monoclonal antibodies are particularly important when maternal vaccination is absent, too close to delivery, or potentially ineffective, or when the infant is preterm or otherwise at increased risk. Surveillance and locally adapted implementation are essential to maintain protection as RSV epidemiology evolves.

## 1. Introduction

Respiratory syncytial virus (RSV) is a leading cause of bronchiolitis and lower respiratory tract infection (LRTI) in young children and a major cause of hospitalization during the first months of life [1,2,3,4,5,6,7,8]. Severe disease is not confined to infants with recognized risk factors; many hospitalizations occur in previously healthy, term-born infants. Prevention during early infancy is therefore a major immunization and public-health priority.

The prevention landscape has changed substantially with the availability of maternal RSVpreF vaccination and long-acting monoclonal antibodies for infants. Both strategies provide passive protection during the period of greatest vulnerability, but they differ in biological mechanism, timing, delivery setting, dependence on placental antibody transfer, duration of protection, and programmatic requirements [9,10,11,12,13,14,15,16,17,18,19,20,21]. These differences have become more important as RSV circulation has become less predictable after the COVID-19 pandemic, with earlier, later, shorter, and out-of-season epidemics reported in multiple settings [7,8].

This document is intentionally focused on maternal RSV immunization and its place within an integrated strategy for protection during early infancy. Epidemiology and seasonality are considered only insofar as they inform the timing, target population, and implementation of maternal vaccination and infant monoclonal antibody prophylaxis. The review therefore emphasizes (i) the biological basis and timing of transplacental protection; (ii) the efficacy, safety, and emerging real-world effectiveness of maternal vaccination; (iii) the comparative strengths and limitations of long-acting monoclonal antibodies; and (iv) the operational, equity, and economic factors that determine how these approaches can be combined in different healthcare settings.

The principal policy question is not whether maternal vaccination or infant monoclonal antibody prophylaxis is universally superior, because direct comparative evidence remains limited, and their performance depends on context. Rather, the objective is to identify the circumstances in which maternal vaccination is likely to provide adequate protection, the situations in which direct infant prophylaxis should be preferred or added, and the surveillance and implementation conditions required to avoid both gaps in protection and unnecessary duplication.

Accordingly, the WAidid Consensus Development Group developed evidence-informed statements and a practical framework that incorporates maternal vaccination status, gestational age, vaccination-to-delivery interval, infant risk, local RSV epidemiology, antenatal care access, product availability, and resource constraints.

## 2. Methods

In January 2026, a Steering Committee convened a multidisciplinary Consensus Development Group (CDG) of 11 experts with clinical and/or research expertise in epidemiology, pediatrics, infectious diseases, vaccinology, and evidence-based methodology. The scope was predefined as RSV prevention during early infancy, with particular emphasis on maternal immunization and its relationship to infant monoclonal antibody prophylaxis. The CDG identified the key domains for evidence review: RSV burden and seasonality relevant to infant protection; maternal RSV vaccination; transplacental antibody transfer and timing; long-acting monoclonal antibodies; real-world effectiveness; implementation and equity; and economic considerations.

A comprehensive literature review was conducted between February and March 2026 using PubMed, Embase, Web of Science, and the Cochrane Library without date restrictions. Medical Subject Headings and free-text terms covered RSV, lower respiratory tract infection, newborns and infants, maternal immunization, pregnancy, monoclonal antibodies, seasonality, effectiveness, implementation, and cost-effectiveness. Reference lists of eligible primary studies and relevant systematic reviews were screened for additional evidence, and searches were updated during manuscript preparation to capture newly available data. Primary studies and current recommendations from major health authorities were prioritized when available; reviews were used mainly to contextualize or identify primary evidence.

Evidence selection and extraction were performed independently by pairs of reviewers. Disagreements were resolved by discussion and, when necessary, consultation with a third reviewer. The evidence was synthesized by domain and used to draft preliminary consensus statements. Because the available evidence includes randomized trials, observational effectiveness studies, surveillance data, and economic models with heterogeneous designs and outcomes, the group did not perform a formal meta-analysis or de novo certainty-of-evidence grading. This limitation is explicitly considered when interpreting comparative statements.

A modified Delphi process was used to establish consensus. All 11 CDG members rated each proposed statement on a five-point Likert scale (strongly agree, agree, neither agree nor disagree, disagree, strongly disagree) and could provide free-text comments. Consensus was prespecified as at least 75% of participants selecting agree or strongly agree. Voting was completed through an online questionnaire in April 2026. Statements not meeting the threshold were not included as consensus recommendations; wording was refined in response to comments, and the final manuscript presents only statements and practical scenarios that achieved the predefined agreement threshold. The consensus framework should therefore be interpreted as expert guidance informed by the reviewed evidence, rather than as a formal guideline based on a GRADE process.

## 3. Epidemiology of RSV

RSV transmission, similarly to that of other respiratory pathogens such as influenza viruses and SARS-CoV-2, occurs mainly through close interpersonal contact and exposure to infectious respiratory droplets and aerosols [22,23,24]. After initial replication in the epithelial cells of the nasopharynx, where infection usually causes mild to moderate upper respiratory tract illness, RSV may spread to the small bronchioles and alveolar spaces, resulting in lower respiratory tract involvement. Pneumonia and bronchiolitis are among the most common clinical manifestations. Bronchiolitis, which predominantly affects young infants, is largely driven by narrowing of the lower airways due to mucus hypersecretion, epithelial injury, and airway inflammation sustained by the host immune response [25,26,27,28].

Although bronchiolitis is not exclusively caused by RSV, it is classically regarded as the clinical syndrome most closely linked to RSV infection [29,30,31]. Nevertheless, most clinical manifestations of RSV infection are non-specific and overlap substantially with those caused by other respiratory viruses. In a comparative analysis by Falsey et al. [32], adult RSV cases showed higher frequencies of nasal congestion, dyspnea, and particularly wheezing than cases of seasonal influenza A infection (57.63% vs. 41.35%, *p* = 0.03; 80.51% vs. 64.66%, *p* = 0.03; and 61.86% vs. 39.85%, *p* = 0.02, respectively). Conversely, extensive and often multifocal pulmonary involvement on computed tomography has been reported as a radiologic feature more strongly associated with RSV than with influenza A [33].

Because of this substantial clinical overlap, definitive diagnosis requires laboratory confirmation through detection of RSV in respiratory specimens. Real-time polymerase chain reaction (RT-PCR) assays provide the highest sensitivity, whereas antigen-based tests offer more rapid but generally less sensitive alternatives [34,35,36,37]. Nasopharyngeal swabs are commonly used in routine practice, although nasal washes and aspirates may provide higher diagnostic sensitivity in selected settings [34,35,36,37].

When diagnostic testing is not performed, a considerable proportion of RSV infections remain unrecognized. In a retrospective analysis of Italian hospital discharge records from 2015 to 2019, 67,746 hospitalizations were considered likely attributable to RSV; however, only 40.1% were correctly coded as RSV, whereas most were classified as acute bronchiolitis without etiologic attribution, despite being highly suggestive of having been caused by RSV [6,38]. Even when RT-PCR is used, suboptimal sampling may lead to missed diagnoses [34,35,39,40,41,42]. In a prospective cohort study from Louisville, Kentucky, RSV was identified in 56 hospitalized patients using nasopharyngeal swabs alone, whereas nearly half of all RSV-positive cases were detected only when additional respiratory specimens were included [41]. These findings support the view that a substantial proportion of incident RSV infections are either undiagnosed or misclassified as other viral respiratory syndromes [43].

Available estimates suggest that RSV may cause up to 33 million episodes of acute respiratory infection and lower respiratory tract infection each year [3,44,45], with a global hospitalization rate estimated at approximately 5.3 per 1000 people per year (95% confidence interval [CI], 4.2–6.8) [2,3,46,47,48,49,50]. However, these estimates do not capture the full outpatient burden, which remains difficult to quantify globally [51]. In the systematic review by Heemskerk et al. [51], annual incidence rates of primary care consultations attributable to RSV varied widely, from 0.8 to 330 per 1000 population. Even among high-income countries, estimates remain highly heterogeneous, largely reflecting differences in healthcare organization, access to primary care, diagnostic practices, and surveillance systems. For example, a recent retrospective study from Germany estimated 1.3 to 3.9 million annual outpatient consultations for RSV across all age groups between 1998 and 2022, with 43.5% occurring in children aged 0–4 years [52]. By contrast, an earlier UK study estimated approximately 350,000 annual medical consultations attributable to RSV in children younger than 5 years [53].

### 3.1. Seasonality of RSV Circulation

RSV has historically shown marked seasonality, although the timing and intensity of epidemics vary by climate and geography. In many temperate Northern Hemisphere settings, activity traditionally increased in late autumn and winter, whereas tropical regions may experience rainy season peaks or year-round circulation [44,45,53,54,55,56,57,58,59,60,61,62,63,64,65,66,67,68,69,70,71,72,73,74]. Consequently, a fixed seasonal schedule has never been equally applicable to all settings.

The COVID-19 pandemic further disrupted these patterns. Following relaxation of non-pharmaceutical interventions, many countries reported unusually early, delayed, short, prolonged, or out-of-season RSV epidemics, and irregular circulation persisted in several settings through 2024 [7,72,75,76,77,78,79,80,81,82,83,84,85,86,87,88,89,90,91,92]. These changes are clinically relevant because infants born outside the historical RSV season may still encounter substantial RSV exposure during the first months of life.

Seasonality instability has different implications for the two prevention modalities. Maternal vaccination must be timed early enough before delivery to permit an adequate maternal immune response and transplacental antibody transfer, while also providing antibodies during the infant’s period of exposure. Long-acting monoclonal antibodies can be administered directly to the infant and therefore offer greater flexibility when birth timing and RSV circulation do not align with a predefined maternal vaccination season. In regions with irregular or year-round circulation, prevention programs may require surveillance-guided timing, broader seasonal windows, or risk-based year-round delivery rather than reliance on historical winter schedules alone.

### 3.2. Burden of RSV in Infants and Children

Nearly half of all infants acquire RSV infection during their first year of life. In 2019, RSV was estimated to cause approximately 33 million episodes of illness in children younger than 5 years worldwide, generating substantial medical, social, and economic burden [3]. Although most infections are mild and managed in primary care, a considerable proportion progress to severe lower respiratory tract disease requiring hospitalization, and some require admission to the pediatric intensive care unit (PICU). Mortality remains a major concern, particularly in low-resource settings where access to timely supportive care may be limited.

The relevance of RSV in primary care has been highlighted by a recent multicountry cohort study conducted across Belgium, Italy, Spain, the Netherlands, and the UK over three consecutive RSV seasons [5]. Among 3414 children younger than 5 years presenting with acute respiratory tract infection, 32.9% tested positive for RSV, with the highest positivity observed among infants. Mean illness duration was 11.7 days, and persistent symptoms were common up to 30 days after disease onset. Healthcare utilization was substantial, with 1.4–3.0 primary care visits per episode and higher visit frequency among infants. The societal burden was also notable, as nearly half of parents reported missed workdays.

Estimates of RSV-associated costs for patients, families, and healthcare systems remain highly heterogeneous, reflecting differences in healthcare organization, reimbursement systems, diagnostic practices, and case definitions. For example, a recent Italian retrospective study estimated mean hospitalization costs ranging from EUR 2007 to EUR 2617 [6]. By contrast, a US study based on a national sample of privately insured children younger than 5 years estimated mean costs of USD 28,586 per episode of RSV-associated LRTI requiring inpatient care, with substantial variability across cases [93].

At the severe end of the disease spectrum, RSV infection in 2019 resulted in an estimated 3.6 million hospitalizations and more than 100,000 deaths globally among children younger than 5 years [3]. Approximately 75% of RSV-associated hospitalizations occur in young infants [94], and nearly 10% of hospitalized children require ICU care [95]. Although severe RSV disease is more frequent in children with underlying conditions, most severe cases occur in previously healthy infants [44,48,51,95,96,97]. Conditions that markedly increase the risk of complications include congenital heart disease, neuromuscular impairment, trisomy 21, bronchopulmonary dysplasia, chronic respiratory disease, and immunosuppression. In selected high-risk populations, case fatality ratios may be substantial; for instance, RSV infection in bone marrow transplant recipients has been associated with a case fatality ratio of 7.28% [98].

A nationwide Swedish cohort further characterized risk factors for severe RSV outcomes [4]. Among children admitted to the ICU, the median age was 1.9 months, and 41.3% had at least one underlying comorbidity. Factors strongly associated with ICU admission or death included birth during winter, small-for-gestational-age status, multiple birth, having young siblings, and the presence of comorbidities. Notably, comorbidities were less frequent among severe cases occurring in infants younger than 3 months, emphasizing the intrinsic vulnerability of this age group. Although most infants who develop severe RSV disease are previously healthy, identifying groups at increased risk morbidity remains essential for prioritizing preventive strategies while broader immunization programs are implemented. Reliable estimates of disease burden and clearly defined risk factors are also critical for policymakers, who must determine the most appropriate and sustainable RSV prevention pathways at the population level.

## 4. Preventive Options

Until recently, prevention of RSV disease relied primarily on palivizumab, a monoclonal antibody targeting antigenic site II of the RSV F protein. Most candidate RSV vaccines had failed to achieve sufficient efficacy, and available prophylaxis was limited to selected high-risk infants [43,99,100,101,102]. Although palivizumab represented an important advance, its use could not be extended to universal infant protection. Clinical studies showed no clear effect on mortality prevention [99,102], and its clinical utility was limited by several practical and pharmacological constraints.

One of the most important limitations of palivizumab is its relatively short elimination half-life. In infants and young children, the mean half-life is approximately 20–24 days, leading to a progressive decline in serum concentrations within a few weeks after administration. As a result, protective antibody levels cannot be maintained with a single dose, and repeated monthly intramuscular injections are required throughout the RSV season. Because the RSV season usually lasts 4–6 months, effective prophylaxis may require up to five consecutive monthly doses. This schedule imposes a substantial burden on families and healthcare systems, particularly for medically fragile infants, and missed or delayed doses may result in subtherapeutic antibody concentrations during periods of high RSV circulation. Repeated administration is also associated with high direct and indirect costs [103,104]. For example, Wick et al. estimated that the mean cost of palivizumab among infants receiving at least one dose during the first year of life was EUR 5435 for the 2015–2019 birth cohorts [105]. Consequently, palivizumab has generally been reserved for a limited subset of high-risk infants, including children born at ≤35 weeks of gestation and aged <6 months at the onset of the RSV season, children aged <2 years requiring treatment for chronic lung disease within the previous 6 months, and children aged <2 years with hemodynamically significant congenital heart disease [11,103,106,107,108,109].

Since 2023, the RSV prevention landscape has changed substantially with the approval of new long-acting monoclonal antibodies and the maternal vaccine in pregnancy. In the case of maternal vaccination, passive protection to infants through transplacental transfer of RSV-neutralizing antibodies. Passive protection in early life, achieved either by maternal vaccination during pregnancy or by direct administration of monoclonal antibodies to the infant, is particularly relevant because the risk of severe RSV complications, including hospitalization and death, is highest during the first 6 months of life. During this period, active pediatric vaccination may not provide sufficiently rapid or robust protection because of immune immaturity and the narrow window between birth and peak susceptibility [110,111,112]. The main preventive options currently available or recently introduced for RSV differ substantially in target population, mechanism of protection, timing of administration, and programmatic implications. A summary of their principal characteristics is provided in Table 1.

Among these interventions, long-acting monoclonal antibodies have had the most immediate impact on infant prevention programs, as they overcome several of the practical limitations that restricted the use of palivizumab.

### 4.1. Monoclonal Antibodies

Long-acting monoclonal antibodies have been developed to overcome the short half-life and repeated dosing requirements of palivizumab, with the goal of providing protection throughout an entire RSV season after a single administration [113]. Several candidates preceded the currently available products. Motavizumab, a second-generation monoclonal antibody derived from palivizumab, showed efficacy but still required repeated administration during the RSV season and was discontinued in 2010 [113,114]. Suptavumab, which targeted antigenic site III of the prefusion F protein, failed to reduce overall RSV hospitalizations or outpatient RSV-associated lower respiratory tract infection (LRTI), partly because of naturally occurring mutations affecting its binding to dominant RSV strains [115].

#### 4.1.1. Nirsevimab

Nirsevimab is a long-acting human IgG1κ monoclonal antibody directed against antigenic site Ø of the RSV prefusion F protein. It contains a triple amino acid YTE substitution in the Fc region, which extends its serum half-life to approximately 68.7–71 days in infants and allows protection throughout a typical RSV season after a single intramuscular dose. Nirsevimab shows dose-proportional pharmacokinetics, rapid intramuscular absorption, and approximately 85% bioavailability. Plasma concentrations decline progressively but remain detectable for several months after administration [43,116,117,118].

Nirsevimab was approved by the European Medicines Agency in October 2022 and was subsequently authorized in the United Kingdom, Canada, and the United States in 2023 [116]. It is indicated for infants aged <8 months who are born during or entering their first RSV season, with a recommended dose of 50 mg for infants weighing < 5 kg and 100 mg for those weighing ≥ 5 kg. A 200 mg dose is recommended for selected high-risk children entering their second RSV season, including those with chronic lung disease or hemodynamically significant congenital heart disease [43,117,118]. In countries where RSV circulation typically occurs from October or November through March, most health authorities recommend administration during birth hospitalization for infants born during the RSV season and shortly before the RSV season for infants born outside the season. Infants born to mothers who received RSV vaccination during pregnancy do not require an additional administration of nirsevimab, unless maternal vaccination occurred within 14 days before delivery or specific clinical circumstances justify additional prophylaxis (e.g., impaired transplacental transfer of maternal antibodies, high-risk health condition) [16,119].

Since regulatory approval, multiple real-world studies from the Northern Hemisphere, particularly from Europe and Spain, have documented the effectiveness of nirsevimab in preventing RSV disease, with the greatest effect observed against severe outcomes [120,121,122,123,124,125,126]. In a study including 141,550 infants, nirsevimab reduced the risk of RSV-LRTI hospitalization by 84.5% (95% CI, 73.6–90.9), ICU admission by 85.9% (95% CI, 13.2–97.7), and need for ventilatory support by 87.1% (95% CI, 70.2–94.4). Effectiveness was also reported against RSV-LRTI visits in primary care and emergency departments, with reductions of 75.8% (95% CI, 40.4–92.7) and 87.9% (95% CI, 70.3–95.1), respectively [13]. A pooled real-world analysis estimated effectiveness against RSV hospitalization at 90.5% (95% CI, 87.1–93.0), exceeding efficacy estimates from randomized controlled trials, with a very low incidence of adverse events [127].

A larger systematic review including 32 cohort and case-control studies from France, Italy, Luxembourg, Spain, and the USA confirmed these findings. In infants aged 0–12 months, nirsevimab was associated with significantly lower odds of RSV-related hospitalization (odds ratio [OR], 0.17; 95% CI, 0.12–0.23), ICU admission (OR, 0.19; 95% CI, 0.12–0.29), and LRTI incidence (OR, 0.25; 95% CI, 0.19–0.33). However, real-world effectiveness varies according to infant age, prematurity, birth weight, timing of administration, and time elapsed since prophylaxis. Effectiveness against hospitalization appears highest among infants younger than 6 months [128]. This has shifted the age distribution of hospitalized cases toward older infants, reflecting enhanced protection of the youngest and most vulnerable children. At the University Hospital of Saint-Étienne, for example, the median age of hospitalized infants increased from 3.5 months during the 2022–2023 season to 6.2 months by the end of 2023 [126].

Protection also appears to wane over time. In a prospective Spanish study including 29,694 nirsevimab-immunized children and 7373 non-immunized controls, effectiveness against primary care events decreased from 69.0% during the first month after administration to 60.9% in the second month, 50.6% in the third month, and 37.5% in the fourth month. Effectiveness against hospitalization remained higher, decreasing from 93.6% at 30 days to 87.6% at 150 days, while protection against ICU admission remained substantial [129]. In a French matched case-control study of infants younger than 12 months hospitalized for RSV-associated bronchiolitis, nirsevimab effectiveness against PICU admission was 69.6%, and effectiveness against admission requiring ventilatory support was 67.2% [130]. Some studies have also reported slightly lower, although still clinically meaningful, effectiveness in preterm or low-birth-weight infants, suggesting that modified dosing strategies may require further investigation in selected high-risk groups [131,132,133,134].

Across clinical trials, nirsevimab has been safe and well tolerated. Rates of serious adverse events were comparable to placebo, most adverse events were mild to moderate, and injection site reactions and fever were infrequent. No increased safety signal has been reported with repeated seasonal dosing. Economic evaluations indicate that nirsevimab is more effective and offers better value than palivizumab, especially in high-risk populations. However, the cost-effectiveness of universal programs depends strongly on acquisition price, with several analyses suggesting that prices around EUR 220–267 per dose are more likely to be cost-effective [135].

Potential limitations to nirsevimab effectiveness include viral escape mutations and development of anti-drug antibodies [136]. Because RSV exhibits substantial genetic variability [61], variants with reduced susceptibility to nirsevimab could theoretically emerge under selective pressure from widespread prophylactic use. To date, however, naturally occurring RSV strains with reduced susceptibility have been rare across geographic regions and seasons [14,15,137]. Post-marketing surveillance and clinical trial data indicate that resistance remains uncommon and largely confined to RSV B strains. In two Phase IIb/III trials, more than 99% of RSV isolates remained fully susceptible to nirsevimab, with no clinically relevant resistance-associated mutations detected among RSV A strains [103]. Only two participants harbored RSV isolates with triple substitutions in antigenic site Ø associated with reduced susceptibility.

Fourati et al. similarly reported no resistance-associated mutations among 472 RSV A strains, approximately half of which were obtained from nirsevimab-treated infants [138]. By contrast, resistance-associated substitutions were identified in 2 of 24 RSV B breakthrough infections, including the previously described F:N208D mutation and a novel combination of F:I64M and F:K65R substitutions [139]. Although the detection of resistant RSV B variants highlights the need for continued surveillance, current evidence indicates that the risk of clinically significant resistance remains very low. This is further supported by epidemiological data showing that RSV A strains predominate in most regions and during most seasons [140].

Anti-drug antibodies have been detected in a subset of children receiving nirsevimab. Available evidence suggests that they have minimal impact on pharmacokinetics and have not been associated with clinically significant safety concerns. However, their potential relevance for repeated dosing warrants further evaluation. At 151 days after administration, neutralizing antibody concentrations in anti-drug antibody-positive children were comparable to levels associated with protection against severe RSV disease. Approximately 1 year after immunization, however, neutralizing antibody concentrations were lower in anti-drug antibody-positive children than in those without detectable anti-drug antibodies [12]. These findings suggest that anti-drug antibodies could attenuate responses to subsequent nirsevimab administration, potentially reducing the effectiveness of second-season dosing in high-risk children.

Beyond RSV-specific outcomes, nirsevimab may reduce broader respiratory morbidity. A meta-analysis of prelicensure trials reported a 54% reduction in all-cause respiratory illness-related hospitalizations among infants aged ≤12 months (risk ratio, 0.46; 95% CI, 0.36–0.59) [141]. A postlicensure meta-analysis including 236,764 infants and children aged ≤24 months in the nirsevimab group and 27,522 controls reported real-world effectiveness of 62% against all-cause LRTI-related hospitalization, 48% against all-cause LRTI-related emergency department visits, and 76% against RSV-related LRTI emergency department visits [142]. This apparent broader effectiveness is likely explained by the large contribution of RSV to medically attended respiratory illness during seasonal epidemics. Studies conducted outside RSV seasons have not shown protection against all-cause LRTI, supporting the interpretation that these effects largely reflect prevention of RSV-associated disease. A secondary contribution from reduced bacterial coinfections after RSV prevention is also biologically plausible.

#### 4.1.2. Clesrovimab

Clesrovimab is a fully human long-acting monoclonal antibody targeting the prefusion conformation of the RSV F protein. Unlike palivizumab and nirsevimab, which bind antigenic sites II and Ø, respectively, clesrovimab binds antigenic site IV. This distinct epitope may offer protection against RSV variants with mutations that reduce susceptibility to other monoclonal antibodies. Its estimated half-life is approximately 44–45 days [143].

Clesrovimab has received regulatory approval for the prevention of RSV-associated lower respiratory tract disease in infants born during or entering their first RSV season [144,145]. Unlike nirsevimab, it is not currently approved for children at increased risk entering their second RSV season. It is administered as a single intramuscular fixed dose of 105 mg, independent of body weight. Clinical trial data suggest that clesrovimab can provide rapid and durable protection for approximately 5 months, corresponding to the typical duration of an RSV season.

Regulatory authorization was supported by two pivotal clinical studies. In a randomized, double-blind, placebo-controlled trial including 3632 healthy preterm and full-term infants aged from birth to 12 months entering their first RSV season, RSV-associated medically attended LRTI through day 150 occurred in 2.6% of infants receiving clesrovimab and 6.5% of those receiving placebo, corresponding to an efficacy of 60.4% (95% CI, 44.1–71.9; *p* < 0.001) [146]. RSV-associated hospitalization occurred in 9 infants in the clesrovimab group and 28 infants in the placebo group, yielding an efficacy against hospitalization of 84.2% (95% CI, 66.6–92.6; *p* < 0.001). Serious adverse events were similar between groups, occurring in 11.5% and 12.4% of infants, respectively.

Preliminary analyses also suggested sustained protection against severe disease, with efficacy against RSV-associated hospitalization due to LRTI estimated at 90.9% at 150 days and 91.2% at 180 days after administration. Similar estimates were reported for medically attended RSV-associated LRTI. A second study evaluated clesrovimab in infants and children at increased risk for severe RSV disease, including those born extremely preterm and those with congenital heart disease or chronic lung disease, using palivizumab as the comparator [147]. Rates of RSV-related illness and hospitalization were similar between groups, and the safety profile was consistent with that observed in the healthy-infant trial. Efficacy in this higher-risk population was supported by pharmacokinetic extrapolation from the healthy infant efficacy study.

Although short-term safety data are reassuring [148], continued post-marketing pharmacovigilance is essential to detect rare adverse events and assess effectiveness in broader real-world populations, including low- and middle-income countries, where comorbidities, access to care, and RSV-associated mortality differ substantially from trial settings.

The clinical role of clesrovimab will also depend on comparative effectiveness and cost-effectiveness data. A mathematical modeling study comparing clesrovimab and nirsevimab found that nirsevimab consistently prevented more RSV medically attended lower respiratory tract disease and generated lower direct and indirect healthcare costs. In that analysis, nirsevimab was dominant, meaning more effective and less costly, across modeled scenarios [21,149]. These findings should be interpreted in the context of evolving prices, local epidemiology, and implementation costs.

#### 4.1.3. Emerging Monoclonal Antibodies

Additional monoclonal antibodies are in development. 5B11 targets a highly conserved and immunologically subdominant epitope within antigenic site V of the RSV F protein. In vitro studies suggest potent neutralizing activity against RSV A and B strains, including nirsevimab-resistant variants such as B18537 [150]. In animal models, 5B11 demonstrated superior protection compared with nirsevimab [151]. However, comprehensive evaluation of safety, tolerability, pharmacokinetics, and clinical efficacy in infants will be required before its potential role in clinical practice can be defined.

TNM001, also known as trinomab, is another monoclonal antibody targeting the RSV prefusion F protein. It is currently being evaluated in a Phase III clinical trial enrolling both preterm and term infants for protection during their first RSV season (NCT06083623). Its clinical performance will help clarify its potential place in the evolving landscape of RSV immunoprophylaxis.

Because most advanced RSV monoclonal antibodies are being developed primarily for high-income markets, anticipated costs may limit global accessibility. Candidate products designed for low-resource settings are therefore of particular interest. SM01 is a fully human IgG1 monoclonal antibody targeting antigenic site Ø of the RSV prefusion F protein and engineered for extended half-life [152]. It has shown potent inhibition of RSV infection and replication in vitro and in vivo, including activity against selected strains with reduced susceptibility to currently licensed antibodies. In a Phase Ia first-in-human study in healthy adults, SM01 was generally safe and well tolerated, with pharmacokinetic data suggesting the potential for season-long protection after a single dose [153]. Further infant studies and scalable, cost-efficient manufacturing will be critical to determine whether this approach can improve access in regions with the highest RSV-associated infant mortality.

### 4.2. Vaccines

Among the many RSV vaccine candidates developed or under evaluation [43,97,154,155,156,157,158,159,160,161,162,163,164,165,166,167,168,169,170,171,172], three are currently approved for use in older adults: the bivalent non-adjuvanted RSVpreF vaccine from Pfizer, the adjuvanted RSVpreF3 vaccine from GSK, and the mRNA-1345 vaccine from Moderna [173,174,175,176,177,178,179,180,181,182,183,184,185,186,187,188,189,190,191,192,193,194,195,196,197,198,199,200,201,202,203,204,205,206]. RSVpreF is also authorized for use during pregnancy to protect infants through transplacental transfer of vaccine-induced antibodies. No RSV vaccine is currently authorized for direct use in infants or children.

Both RSVpreF and RSVpreF3 are protein-based subunit vaccines targeting the prefusion conformation of the RSV F protein. However, they differ in formulation. RSVpreF is a bivalent, non-adjuvanted vaccine containing equal amounts of prefusion F antigen from RSV A and RSV B strains, whereas RSVpreF3 is a monovalent RSV A prefusion F vaccine formulated with the AS01 adjuvant [203,206]. The mRNA-1345 vaccine contains a single mRNA sequence encoding a stabilized RSV A prefusion F protein and thus does not contain an adjuvant [161,180]. Current schedules for these vaccines are based on single-dose administration, and routine annual revaccination is not presently recommended.

Recommendations in the USA and several European countries generally include vaccination of adults aged ≥75 years and adults aged 50–74 years with underlying conditions that increase the risk of severe RSV disease, such as chronic respiratory or cardiovascular disease. Adults aged ≥60 years living in long-term care facilities are also commonly included in vaccination recommendations. The following sections summarize available evidence for adult vaccination and maternal vaccination.

#### 4.2.1. Maternal Vaccination

Maternal vaccination is an established strategy for preventing severe infectious diseases during early infancy, a window of high susceptibility to severe infection, and when active infant immunization may be limited by immune immaturity. Maternal immunization against tetanus, pertussis, influenza, and COVID-19 provides the main programmatic precedent for this approach [182]. For RSV, the rationale was initially supported by evidence showing efficient transplacental transfer of naturally acquired RSV-neutralizing antibodies [183,184] and vaccine-induced antibodies, including in low-birth-weight neonates [185].

To date, only RSVpreF-based vaccination has received regulatory approval for use during pregnancy from agencies such as the FDA, EMA, and Health Canada [186]. The mRNA-1345 vaccine remains under evaluation in pregnant individuals, while safety concerns have limited development of RSVpreF3 for maternal immunization. In a Phase III trial of RSVpreF3, an increased risk of preterm birth was observed in the vaccine group compared with controls, prompting suspension of enrollment in an ongoing trial [187,188]. A numerical imbalance in preterm births was also observed in a trial of RSVpreF administered between 24 and 36 weeks of gestation, although the finding was not considered conclusive because of the limited number of events, geographic differences (observed in low-middle income countries and not in high-income countries), and possible confounding by the SARS-CoV-2 Delta wave [189]. A subsequent Cochrane review of six randomized controlled trials including 17,560 infants suggested that maternal RSV vaccination may be associated with a slight increase in preterm birth, but the certainty of evidence was rated very low [188].

To mitigate potential risk, the FDA and ACIP recommend administration of RSVpreF between 32 and 36 weeks of gestation and during the RSV season, typically from September to January in the USA [188,190,191]. Similar guidance has been adopted in Canada and several South American countries, including Argentina [192,193,194]. In contrast, the EMA endorses a broader window from 24 to 36 weeks of gestation [193,194]. The UK Joint Committee on Vaccination and Immunisation and the Belgian Superior Health Council recommend vaccination after 28 weeks of gestation, citing uncertainty around earlier administration [195,196]. Further clarification of these safety signals is essential, because a wider vaccination window could improve coverage, particularly in settings where access to prenatal care is inconsistent. Additional evaluation is also needed for other potential adverse events, including hypertensive disorders of pregnancy and Guillain–Barré syndrome, for which current evidence remains limited.

Beyond safety, timing is a critical determinant of maternal vaccine effectiveness. Jasset et al. showed that vaccination 2–3 weeks or 3–4 weeks before delivery was associated with significantly lower cord-to-maternal antibody transfer ratios than vaccination more than 5 weeks before delivery [197]. Cord-to-maternal ratios were also lower when vaccination occurred 2–4 weeks before delivery compared with more than 6 weeks before delivery. Although maternal antibody concentrations did not differ significantly according to the vaccination-to-delivery interval, these findings suggest that vaccination at least 5 weeks before delivery may optimize transplacental antibody transfer. This observation has important implications for programs using a narrow 32–36 week window, particularly for pregnancies at risk of earlier delivery. The effectiveness of maternal RSV vaccination program in routine practice is determined not only by vaccine efficacy, but also by biological, obstetric, epidemiological, and health system factors that influence antibody transfer, infant exposure, and program uptake. These determinants are summarized in Table 2.

These considerations are particularly important because maternal vaccination aims to provide protection during the earliest months of life, when the risk of severe RSV disease is greatest.

Maternal RSV vaccination can provide protection during the first months of life. In a randomized controlled trial, RSVpreF efficacy against RSV-associated LRTI was 57.1% at 90 days after birth, 56.8% at 120 days, 52.5% at 150 days, and 51.3% at 180 days. Efficacy against medically attended severe LRTI ranged from 81.8% at 90 days to 69.4% at 180 days, while efficacy against RSV-associated hospitalization was 67.7% at 90 days and 56.8% at 180 days [17]. A Japanese subset analysis reported efficacy against medically attended RSV-LRTI of 100% during the first 90 days and 87.6% during the first 180 days, although the small sample size limited precision for severe outcomes [191].

Real-world evidence has reinforced these clinical trial findings. The BERNI study, a multicenter retrospective test-negative case-control study conducted in Argentina during the 2024 RSV season, evaluated infants born to individuals vaccinated with RSVpreF between 32+0/7 and 36+6/7 weeks of gestation, with vaccination at least 14 days before delivery [18]. Among 633 infants hospitalized for LRTD, 18% of RSV cases and 50% of controls were born to vaccinated mothers. Vaccine effectiveness against RSV-associated LRTD hospitalization was 78.6% from birth to 3 months and 71.3% from birth to 6 months. Effectiveness against severe RSV-associated LRTD requiring hospitalization was 76.9% from birth to 6 months. Notably, all three RSV-associated in-hospital deaths occurred in infants born to unvaccinated mothers. Additional analyses showed that effectiveness was maintained when RSVpreF was co-administered with other pregnancy vaccines, supporting integration into antenatal immunization programs. Similar real-world findings have been reported in the USA and Australia [198,199].

The impact of maternal vaccination will ultimately depend on coverage. Available studies suggest generally favorable acceptance of maternal RSV immunization, with willingness ranging from 54% to 89% across settings [19]. Acceptance is driven primarily by the desire to protect the newborn from severe respiratory disease, but varies by region, with higher acceptance reported in Canada and Australia and lower acceptance in some European countries [19]. Strong healthcare provider recommendation, confidence in safety, convenience, and the possibility of administration during routine antenatal visits are among the most important determinants of uptake.

Available studies suggest generally favorable acceptance, with willingness ranging from 54% to 89% across settings [19]. Strong healthcare provider recommendation, confidence in safety, convenience, and administration during routine antenatal visits are important determinants of uptake. Protection is greatest in the first months of life and decreases as maternally derived antibody concentrations decline; therefore, infants whose exposure occurs substantially later after birth may have less residual protection. This waning is particularly relevant for infants born outside conventional RSV seasons or in settings with unpredictable circulation and reinforces the need to coordinate maternal programs with infant prophylaxis pathways.

#### 4.2.2. Integrated Prevention Strategies for Infants and Young Children

Maternal RSV vaccination and long-acting monoclonal antibody prophylaxis should be evaluated as two distinct routes to the same immediate objective: passive protection during early infancy. Maternal vaccination generates antibodies that must be transferred across the placenta, whereas monoclonal antibodies are administered directly to the infant. The interventions therefore differ in onset, determinants of effectiveness, operational platform, duration of protection, and cost. Table 3 summarizes these differences and provides the basis for a context-specific rather than competitive interpretation of the two strategies.

#### 4.2.3. Comparative Effectiveness and Duration of Protection

No randomized head-to-head trial has established the superiority of maternal RSVpreF vaccination over long-acting monoclonal antibodies or vice versa. Cross-trial comparisons are inherently limited by differences in populations, endpoints, follow-up, RSV seasons, and healthcare systems. Maternal vaccination has demonstrated protection against medically attended RSV-associated LRTI, severe disease, and hospitalization during early infancy, whereas nirsevimab has shown high effectiveness against hospitalization and severe outcomes in multiple post-licensure studies. Clesrovimab has demonstrated efficacy in clinical trials, but real-world experience is more limited. Policy comparisons should therefore distinguish trial efficacy from real-world effectiveness and avoid treating estimates generated in different study designs as directly interchangeable [200,201,202,203].

The emerging comparative evidence is observational. A recent French study comparing infants whose mothers received RSVpreF vaccination at 32–36 weeks of gestation with infants who received nirsevimab at birth reported fewer RSV-associated LRTI hospitalizations and severe outcomes in the nirsevimab group [204]. This finding is relevant but cannot establish causal superiority, because treatment selection, timing, birth characteristics, healthcare-seeking behavior, and residual confounding may differ between groups. Further prospective comparative studies across complete birth cohorts and multiple RSV seasons are needed.

Duration and timing are central to interpretation. Maternal protection is present from birth when vaccination occurs sufficiently before delivery but depends on maternal response, placental transfer, gestational age, and subsequent antibody waning. Long-acting monoclonal antibodies provide rapid direct protection and are less affected by prematurity or maternal immune status, although their effectiveness also declines over time. The practical advantage of either approach therefore varies with the interval between immunization and RSV exposure rather than with a single efficacy estimate.

#### 4.2.4. Seasonality, Birth Cohorts, and Complementary Use

Unstable RSV seasonality increases the risk that infants born outside conventional seasonal windows will be exposed when maternally derived antibodies have waned or when a maternal seasonal campaign was not offered. A complementary strategy can reduce this vulnerability: maternal vaccination may provide protection from birth for infants whose mothers are vaccinated sufficiently before delivery, while infant monoclonal antibody prophylaxis can provide direct protection when maternal vaccination was not given, occurred too close to delivery, is expected to generate an inadequate response, or does not adequately cover the infant’s anticipated period of RSV exposure. In settings with irregular or prolonged circulation, locally defined broader windows or surveillance-guided infant administration may be preferable to a rigid calendar-based approach.

This complementarity does not imply routine dual administration to all infants. Most term infants born to appropriately vaccinated mothers are expected to be adequately protected without an additional monoclonal antibody. Additional infant prophylaxis is most defensible when the probability or duration of maternally derived protection is reduced, including birth less than 14 days after maternal vaccination, prematurity, maternal immunocompromising conditions, impaired placental transfer, or infant conditions associated with a high risk of severe RSV disease. This approach seeks to avoid both under-protection and unnecessary duplication.

#### 4.2.5. Operational Feasibility, Equity, and Cost-Effectiveness

The preferred programmatic strategy depends on the delivery platform. Maternal vaccination can leverage established antenatal services and may be attractive where prenatal care coverage is high and vaccine delivery during pregnancy is reliable. Infant monoclonal antibody programs may provide more dependable coverage where antenatal access is fragmented, vaccination is frequently missed, delivery timing is unpredictable, or direct administration during the birth hospitalization can achieve high uptake. Equity considerations are therefore not ancillary: a strategy that performs well in trials may produce limited population benefit if the target population cannot reliably access the relevant care pathway.

Economic models reach different conclusions because the results are highly sensitive to product acquisition prices, RSV incidence, hospitalization costs, coverage assumptions, duration of protection, and whether caregiver productivity losses are included [20,21,205,206]. Some models favor universal monoclonal antibody use, whereas others identify mixed maternal-plus-targeted-infant strategies as economically attractive. These analyses should inform, rather than determine, policy. Local price, infrastructure, epidemiology, and achievable coverage must be considered alongside clinical effectiveness.

#### 4.2.6. Consensus Framework for Early Infancy RSV Prevention

On the basis of the reviewed evidence and the modified Delphi process, the CDG agreed on the practical scenarios summarized in Table 4. All statements included in the framework met the prespecified consensus threshold of at least 75% agreement. The framework is designed to identify the most appropriate route to passive protection according to maternal vaccination status, vaccination-to-delivery interval, gestational age, infant risk, anticipated RSV exposure, healthcare access, and local program constraints.

The framework supports four actionable principles. First, maternal vaccination alone is generally appropriate for term infants when vaccination occurred sufficiently before delivery and no condition is expected to impair maternal response or placental transfer. Second, direct infant monoclonal antibody prophylaxis should be used when maternal vaccination was not administered, occurred too close to delivery, or is unlikely to provide adequate protection and should be considered for preterm and other high-risk infants. Third, seasonal timing should be adapted to local surveillance; regions with irregular or prolonged RSV circulation may require broader or year-round risk-based approaches. Fourth, national programs should select the balance between maternal and infant delivery according to achievable coverage, equity, product availability, and cost, with surveillance used to identify protection gaps and update policy.

## 5. Conclusions

This consensus document defines maternal RSV immunization as one component of an integrated early infancy prevention strategy rather than as an isolated intervention. Maternal RSVpreF vaccination can protect infants from birth, but its effectiveness depends on vaccination timing, maternal immune response, placental transfer, gestational age, uptake, and the interval between birth and RSV exposure. Long-acting monoclonal antibodies provide direct protection independent of these maternal determinants and are therefore particularly important when maternal protection is absent, delayed, uncertain, or insufficient.

Current evidence does not support a universal hierarchy between maternal vaccination and infant monoclonal antibodies. Direct comparative evidence remains limited, and emerging observational studies should be interpreted cautiously. The most appropriate strategy is context-dependent: reliable antenatal systems may support maternal vaccination-centered programs with targeted infant prophylaxis, whereas settings with poor antenatal access, unpredictable delivery timing, or unstable RSV circulation may achieve more consistent protection through infant-centered or mixed approaches.

The central implementation objective is continuous protection during the period of greatest infant vulnerability while avoiding unnecessary duplication. Programs should therefore link obstetric and pediatric records, document maternal vaccination status and timing, identify infants requiring monoclonal antibody prophylaxis, and use local surveillance to adapt seasonal windows. Equity, operational feasibility, and product cost should be considered alongside efficacy and effectiveness.

Priority evidence gaps include prospective head-to-head effectiveness studies, evaluation across complete birth cohorts and irregular RSV seasons, duration of protection after maternal vaccination and monoclonal antibodies, effectiveness in preterm and medically vulnerable infants, resistance surveillance, and comparative economic analyses using real-world coverage and pricing. These data will be necessary to refine the balance between maternal and infant immunization as the prevention landscape evolves.

## Figures and Tables

**Table 1:** Currently available RSV preventive options and their main characteristics.

Preventive Option	Target Population	Mechanism of Protection	Timing/Schedule	Main Strengths	Main Limitations/Implementation Issues
Palivizumab	Selected high-risk infants and young children	Passive immunization through monoclonal antibody binding to antigenic site II of the RSV F protein	Monthly intramuscular injections during the RSV season, usually up to five doses	Established safety experience; option for selected high-risk children	Short half-life; repeated dosing; high costs; logistical burden; not suitable for universal infant protection
Nirsevimab	Infants entering their first RSV season; selected high-risk children entering their second season	Long-acting monoclonal antibody targeting antigenic site Ø of the prefusion RSV F protein	Single intramuscular dose before or during RSV season; dose based on body weight	High effectiveness against RSV-associated hospitalization, ICU admission, and severe LRTI; single-dose administration	Cost-effectiveness depends on price; effectiveness may decline over time; continued surveillance needed for resistance and anti-drug antibodies
Clesrovimab	Infants born during or entering their first RSV season	Long-acting monoclonal antibody targeting antigenic site IV of the prefusion RSV F protein	Single fixed intramuscular dose	Single-dose protection; distinct binding site may be useful against some variants	Limited real-world experience; not approved for second-season high-risk children; comparative effectiveness and cost-effectiveness require further evaluation
Maternal RSVpreF vaccination	Pregnant individuals, to protect infants after birth	Active maternal immunization leading to transplacental transfer of RSV-neutralizing antibodies	During second or third trimester of pregnancy; recommended window varies across regulatory authorities and countries	Protects newborns from birth; can be integrated into antenatal care; reduces medically attended RSV-LRTI, severe LRTI, and hospitalization	Effectiveness depends on timing before delivery, placental transfer, prematurity, maternal immune response, and uptake
Adult RSV vaccines	Older adults and adults with risk factors for severe RSV disease	Active immunization against prefusion RSV F protein	Single dose; revaccination interval not yet fully defined	Reduces RSV-LRTD, hospitalization, and severe disease in older adults	Duration of protection, revaccination strategy, safety surveillance, and effectiveness in immunocompromised individuals require further study

**Table 2:** Maternal RSV vaccination: key determinants of effectiveness and programmatic success.

Determinant	Relevance for Maternal RSV Vaccination	Practical Implication
Gestational age at vaccination	Determines the time available for maternal antibody induction and placental transfer	Vaccination too close to delivery may reduce antibody transfer to the infant
Interval between vaccination and delivery	Antibody transfer appears more efficient when vaccination occurs several weeks before birth	Programs should avoid very late vaccination when possible, especially in pregnancies at risk of preterm delivery
Prematurity	Preterm birth shortens the interval for antibody transfer and may reduce infant protection	Preterm infants may require monoclonal antibody prophylaxis even if maternal vaccination was planned or administered
Maternal immune response	The magnitude of maternal antibody production influences antibody levels transferred to the infant	Immunocompromised pregnant individuals may generate lower antibody responses
Placental transfer efficiency	Conditions affecting placental function may reduce antibody transfer	Infants born to mothers with impaired antibody transfer may require additional protection
RSV seasonality	Determines when infants are likely to encounter RSV after birth	Vaccination programs should be aligned with local RSV circulation, especially where seasonality is predictable
Post-pandemic irregular RSV circulation	RSV seasons have become earlier, shorter, delayed, or less predictable in many settings	Flexible strategies may be needed rather than strict reliance on historical RSV seasons
Antenatal care access	Vaccine delivery depends on contact with healthcare services during pregnancy	Integration into routine antenatal visits is essential for high uptake
Healthcare provider recommendation	Strong provider recommendation is one of the most important drivers of maternal vaccine acceptance	Training and communication tools for obstetricians, midwives, and primary care providers are critical
Coordination with infant monoclonal antibodies	Maternal vaccination and infant mAbs may be alternative or complementary depending on context	Clear algorithms are needed to avoid both gaps in protection and unnecessary duplication

**Table 3:** Maternal RSV vaccination versus infant monoclonal antibody prophylaxis.

Feature	Maternal RSV Vaccination	Infant Monoclonal Antibody Prophylaxis
Primary target	Pregnant individual, to protect the newborn	Infant directly
Type of protection	Passive infant protection through transplacental transfer of maternal antibodies	Passive infant protection through administered monoclonal antibody
Onset of infant protection	Present at birth if vaccination occurred sufficiently before delivery	Rapid after administration
Dependence on placental transfer	Yes	No
Impact of preterm birth	May reduce protection because of shorter transfer time	Less affected, because antibody is administered directly to the infant
Dependence on maternal immune response	Yes	No
Programmatic setting	Antenatal care	Birth hospitalization, pediatric care, or seasonal immunization clinics
Best suited for	Term infants born to vaccinated mothers with adequate vaccination-to-delivery interval	Infants born too soon after maternal vaccination, preterm infants, infants at high risk, or settings prioritizing direct infant protection
Main advantages	Protects from birth; leverages antenatal care; may be less costly than universal mAb programs depending on price and uptake	High direct effectiveness; can protect infants regardless of maternal vaccination status or placental transfer
Main limitations	Reduced effectiveness if given too close to delivery; lower protection in some preterm infants; dependent on maternal uptake	Higher product cost; requires infant-level delivery infrastructure; access and affordability may limit universal use
Potential role in combined strategies	Baseline protection for infants born to vaccinated mothers	Additional protection for infants with inadequate maternal protection or high-risk conditions

**Table 4:** WAidid consensus framework for RSV prevention in early infancy.

Clinical or Programmatic Scenario	Preferred/Suggested Strategy	Rationale
Term infant born to a mother vaccinated sufficiently before delivery	Maternal vaccination alone may be adequate	Adequate time for maternal antibody generation and transplacental transfer should provide early-life protection
Infant born <14 days after maternal vaccination	Infant monoclonal antibody prophylaxis	Insufficient time for maternal immune response and antibody transfer
Preterm infant	Consider infant monoclonal antibody prophylaxis, regardless of maternal vaccination status	Shortened gestation may reduce transplacental antibody transfer
Infant born to an unvaccinated mother during or shortly before RSV season	Infant monoclonal antibody prophylaxis	Provides direct and rapid protection during the period of highest risk
Infant born outside the RSV season but entering first RSV season at young age	Seasonal infant monoclonal antibody prophylaxis or maternal vaccination in future pregnancies	Protection should be timed to anticipated RSV exposure
Maternal immunocompromising condition	Infant monoclonal antibody prophylaxis may be preferred or added	Maternal vaccine response may be reduced
Conditions potentially impairing placental antibody transfer	Infant monoclonal antibody prophylaxis may be preferred or added	Infant antibody levels may be insufficient despite maternal vaccination
Infant with high-risk conditions for severe RSV disease	Infant monoclonal antibody prophylaxis; second-season prophylaxis when indicated	Direct protection is warranted because of increased risk of severe outcomes
Settings with reliable antenatal care access and constrained mAb supply	Maternal vaccination-centered strategy with targeted mAb use	May maximize coverage while preserving mAbs for infants unlikely to be protected by maternal vaccination
Settings with poor antenatal vaccine access or unpredictable delivery timing	Infant monoclonal antibody-centered strategy	Direct infant administration may provide more reliable protection
Regions with irregular RSV seasonality	Flexible timing, local surveillance-guided strategy, or year-round risk-based approach	Historical seasonal windows may no longer reliably predict RSV exposure

## Data Availability

No new data were created or analyzed in this study.
